# Consumers’ Perspectives on Eggs from Insect-Fed Hens: A UK Focus Group Study

**DOI:** 10.3390/foods10020420

**Published:** 2021-02-14

**Authors:** Sabrina Spartano, Simona Grasso

**Affiliations:** 1 Department of Applied Economics and Marketing, School of Agriculture, Policy and Development, University of Reading, Reading RG6 6EU, UK; sabrina.spartano@hotmail.it; 2 School of Agriculture, Policy and Development, Institute of Food, Nutrition and Health, University of Reading, Reading RG6 6EU, UK

**Keywords:** animal welfare, circular economy, consumer acceptance, consumer attitudes, food waste, insects as feed, Nvivo, poultry, qualitative study, sustainability

## Abstract

In recent years, there has been growing interest in insects as an alternative to soybean meal as laying hen feed due to nutrition, sustainability, and animal welfare benefits. Although some studies have investigated consumer acceptance and intentions towards insect-fed foodstuffs, no studies are available on eggs from insect-fed hens. This qualitative study aimed to explore consumers’ attitudes and perceptions towards eggs from insect-fed hens and factors influencing intentions to consume and purchase the product. Three focus group discussions were employed with a total of 19 individuals from the UK. Results showed that the environmental, animal welfare, and food waste benefits of feeding hens with insects positively influenced attitudes. Results also indicated price and disgust towards insects as feed were the main barriers, while enhanced welfare standards (e.g., free-range labelling) and information on benefits were main drivers. Therefore, the study suggests that educating and informing consumers about the benefits of feeding hens with insects may increase intentions to consume and purchase eggs from insect-fed hens. Given this emerging area of research, this study contributes to the limited literature on insect-fed foodstuffs and paves the way for further research on the topic.

## 1. Introduction

In the last decade, edible insects have received growing interest as a sustainable alternative source of protein, as both food and animal feed, because of their environmental, nutritional, and animal welfare benefits [1]. In the context of poultry farming and egg production, the use of insects reduces the environmental burden associated with producing traditional feed such as soya, utilises food that would otherwise go to waste, and increases welfare by encouraging natural behaviour without affecting egg quality or taste [2].

Eating insects is a natural behaviour for chickens. When raised in a natural or semi-natural environment (e.g., free-range), chickens spend part of their time foraging and eating insects [2,3].

The European Union has allowed the use of live insects as feed for poultry since 2017 [4]. Although some insect-fed animal foodstuffs have entered the European market (e.g., Oerei eggs in the Netherlands), these products are still considered niche [5]. In the UK, there is a great deal of interest from scientists and companies to produce insects for animal feed. The UK thus represents a potential market for insect-fed eggs and other animal products, although there will likely be a need for regulatory clarification in a post-EU environment [6].

In recent years, consumers have shown increasing acceptance for insect-fed foodstuffs, in particular for insect-fed fish and chicken [7,8,9,10,11,12,13,14,15]. However, given the novelty of insects as feed, it is perhaps unsurprising that research in this area is limited. The available literature embodies mainly studies on insect-fed fish [16,17,18,19,20], studies on insects as feed in general [8,14], and only a few studies that considered insect-fed chickens as a part of broader research [7,9,10,12,13,14].

Bazoche and Poret [20] found neophobia and disgust as some of the main barriers to consumer acceptance of insect-fed animals (aquaculture). This French study showed that while less neophobic consumers were more likely to accept insects as feed, part of the sample appeared disgusted by the idea of eating insect-fed fish. This result is in line with the study of Szendrő, Nagy, and Tóth conducted in Hungary [14]. Despite disgust and neophobia negatively influencing acceptance and intentions, studies agreed that this effect appeared to be less strong for insects as feed than as food [8,13] and could be overcome by informing consumers about the benefits of eating insect-fed foodstuffs [13,14,15,20].

Based on the study of Szendrő, Nagy, and Tóth [14], knowledge of the enhanced animal welfare associated with insect-fed animals may increase its acceptance by Hungarian consumers. Furthermore, as stated by Roma, Palmisano, and De Boni [13] and Bazoche and Poret [20], information on environmental benefits may increase positive intentions among Italian and French consumers. Likewise, Naranjo-Guevara et al. [15] mentioned the importance of providing information not only about environmental benefits but also about the enhanced nutritional content of insects as food and feed.

Among determinants of consumer acceptance, the study of Popoff, MacLeod, and Leschen [17] showed that among UK consumers, nutritional content and taste of insect-fed salmon were more important than price. Conversely, Mancuso, Baldi, and Gasco [16] suggested safety requirements and price as the main factors affecting acceptance of insect-fed fish among Italian consumers. Price also appeared to be an important factor in other studies. According to Bazoche and Poret [20], French consumers were not willing to pay a premium price for insect-fed trout. Similar results were shown by Popoff, MacLeod, and Leschen [17], confirming that although UK consumers considered quality important, they were not willing to pay more for insect-fed salmon. This result was further confirmed by the study of Ankamah-Yeboah, Jacobsen, and Olsen [18], showing that Danish consumers had greater intentions to purchase insect-fed fish at a lower price. In contrast with the aforementioned studies, Ferrer Llagostera et al. [19] showed that Spanish consumers were more likely to purchase insect-fed fish at a premium price.

Although the available research provides insight into consumer acceptance of insects as feed and insect-fed foodstuffs, to the best of our knowledge, there is no available research on consumers’ attitudes and acceptance of eggs from insect-fed hens.

This study is the first one to explore through a qualitative approach consumers’ attitudes and perceptions towards eggs from insect-fed hens and factors influencing intentions.

## 2. Materials and Methods

### 2.1. Focus Groups

Due to the uniqueness of the product under study, the novel topic, and the limited knowledge that consumers have, a qualitative approach based on focus groups was used for the study. This methodology is particularly appropriate in marketing and consumer studies where nothing or little is known about a topic. Focus group discussions provide a more natural environment where respondents share ideas as in real-life situations, making it possible to examine consumers’ perceptions, thoughts, feelings, and beliefs [21,22].

Numerous consumer studies have adopted qualitative research as a data collection method to provide insights into consumers’ motivations, acceptance of, and intentions towards insects as food [23,24,25,26,27,28,29,30,31]. However, to the best of our knowledge, no focus group studies are available on consumer acceptance of insect-fed foodstuffs. Therefore, the aim of the focus groups for this study was to generate insights into the diversity of consumers’ attitudes, opinions, and perceptions towards eggs from insect-fed hens.

### 2.2. Participants

Three focus groups were conducted in the UK in June 2020. Due to recent COVID-19 developments, face-to-face focus groups were replaced with online focus groups undertaken throughout the online platform Zoom, adapting the methodology to the new study design.

A sample of 19 participants (9 male and 10 female) aged from 18 to 56 was recruited and then divided into three groups based on age and gender (see Table 1). Each group participated in a discussion session lasting around 125 min with a 10-min break. Focus group interviews were audio and video recorded.

Participants were recruited utilizing a non-stochastic sampling and then a snowball sampling technique. The recruitment was carried out throughout a short questionnaire posted on social media, and only participants with eligible criteria were selected. Based on inclusion and exclusion criteria, participants were required to be 18 or over, currently living in the UK for at least 6 months, at least partially responsible for food purchase in their household, egg eaters, and egg buyers.

Before the starting date, ethical clearance was obtained by the University of Reading (UK).

### 2.3. Focus Group Structure

A semi-structured protocol with open ended and follow up questions was used to encourage spontaneous answers from participants. The discussion was organized into five main sections and different sub-sections (Table 2).

Section 1: Participants were welcomed on the online platform and introduced to the research project and participation rules.

Section 2: Participants were asked to discuss their egg consumption patterns and attributes they look at when they buy eggs. This stage aimed to identify consumers’ egg preferences in terms of product features and explore the reasons behind their choices.

**Figure 1:**
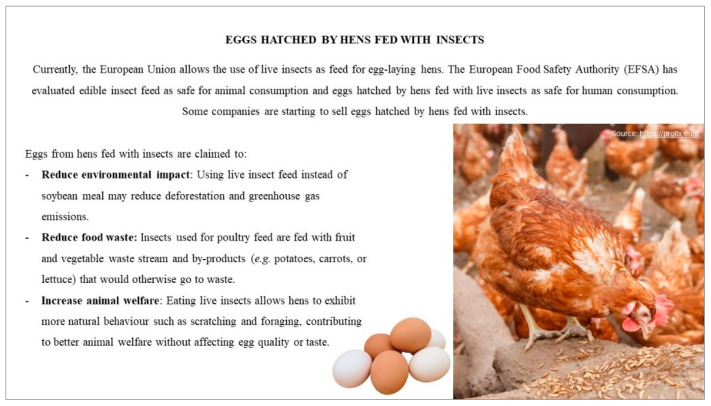
Information card provided to participants during Section 3 of the focus group.

Section 3: Participants were provided with an information card about the benefits of feeding hens with insects (Figure 1). The provision on the information card allowed participants to generate and explore knowledge and opinions about a new product that was not yet in the UK market. After the provision of the information, participants were asked to express their perceptions towards the product and related benefits to identify their level of acceptance.

Section 4: Participants were provided with a translated packaging design of insect-fed eggs that is currently available on the Dutch market. After the provision of the packaging, participants were asked to share opinions about the packaging. The main reason was to understand how packaging information and design may affect consumer perceptions and intentions.

Section 5: Participants were asked to express their intention to consume and purchase the product. This question was meant to explore how perceptions and attitudes towards the product, as well as interest in the benefits, may affect intentions.

At the very end of the discussion, participants were thanked for their participation and their useful contribution.

### 2.4. Data Analysis

Data analysis was carried out on the software Nvivo v. 12 [33]. Audio and video recordings of the focus groups were transcribed and coded using a thematic analysis that is particularly effective to identify and describe themes within a dataset and find patterns among the sample [34].

The qualitative approach used for the analysis incorporated both inductive and deductive methodologies [35]. For this purpose, at first, data were organized in common themes and sub-themes by coding. Themes were generated based on the research questions and recorded under five different nodes. Inductive codes were generated by looking at data and by identifying possible recurring topics. Participants’ quotations belonging to the same themes were codified and recorded under the same node [36]. Each node was successively ramified in sub-nodes, allowing a better distribution and classification of themes and sub-themes. The validity of themes and codes was double-checked by a second researcher. The literature review was used deductively to answer the research questions.

Finally, project maps were used to visualize themes and code relationships. Direct quotes from participants and the names of participants on the project maps are presented with a number (n.1–n.19), gender identification (M or F), and age group (e.g., 18–24 years old).

## 3. Results

Following the focus group sub-sections, results are serially presented as such: (1) egg attributes preferred and related motivations; (2) attitudes and perceptions towards eggs from insect-fed hens; (3) factors influencing attitudes towards eggs from insect-fed hens; (4) reactions and opinions towards the packaging; (5) willingness to buy.

### 3.1. Egg Attributes Preferred and Related Motivations

For most participants, the free-range label (11 cases) was the first attribute they looked at when buying eggs. However, for some of them, organic was always their first choice (6 cases). As a motivation driving them to free-range and/or organic eggs, participants mainly mentioned better animal welfare, better taste, better egg quality, higher nutritional content, and high-quality feed.

The second most important attribute participants looked at when buying eggs was the price (8 cases). While some participants (2 cases) declared they always chose the cheapest eggs, for other participants (5 cases) budget constraints usually drove them to choose a cheaper choice than free-range and organic eggs.

**Figure 2:**
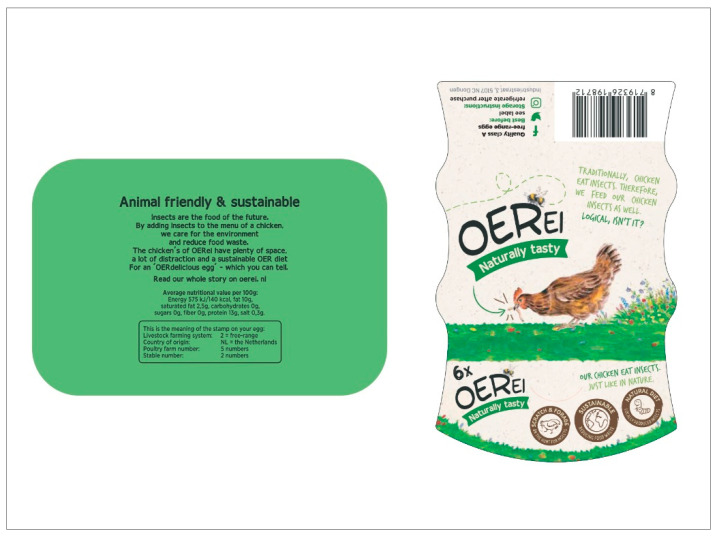
Packaging provided to participants during Section 4 of the focus groups (source: https://oerei.nl/ (accessed on 12 February 2021)).

Other attributes frequently mentioned were size (7 cases), local (6 cases), eco-friendly eggs (6 cases), specialized eggs (4 cases), not broken (4 cases), and British Lion mark (food safety standard) (3 cases) (Figure 2).

### 3.2. Attitudes and Perceptions towards Eggs from Insect-Fed Hens

The majority of participants showed positive attitudes towards eggs hatched by hens fed with insects. In particular, the benefits provided by the products in terms of higher animal welfare, reduction of environmental impact, contribution to reducing food waste, and the perception of a natural method played an important role in increasing intentions to consume and purchase. However, some of the participants revealed a lack of trust in the product (4 cases). They seemed suspicious about the benefits provided and producers’ aims (Table 3).

### 3.3. Factors Influencing Attitudes towards Eggs from Insect-Fed Hens

Other factors that might encourage/discourage participants to consume and purchase the product were mainly price (19 cases), quality and quantity of information provided on the packaging (7 cases), availability (5 cases), nutritional content (5 cases), and taste (4 cases). The feeling of disgust and the rejection of insects seemed to slightly moderate consumers’ intentions. However, only one participant revealed that the negative feeling for insects reduced their level of acceptance (Table 4).

### 3.4. Reactions and Opinions towards the Packaging

All participants showed positive reactions towards the packaging design. Participants mentioned that the box was very “engaging” and immediately brought them to the concepts of “sustainability”, “welfare”, and “natural”. Even though these participants showed an intention to try, the word “insects” written on the packaging would discourage some participants to purchase the product (4 cases). The word “natural”, instead, would encourage some participants to try the product (3 cases).

All participants mentioned the importance of the free-range label on the packaging (19). Everyone agreed that to effectively improve animal welfare, eggs hatched by hens fed with insects should be produced according to high animal welfare standards, and thus, these eggs should be marketed with a free-range label on the packaging.

Again, participants mentioned the importance of clearer information on the packaging (6 cases) (Table 5).

### 3.5. Willingness to Buy for Insect-Fed Eggs

Most participants revealed they were willing to buy the product (18 cases). However, they required knowing the price before making any choice.

Only one participant showed less willingness to buy (1 case) due to the perceived negative feeling towards insects (Table 6).

## 4. Discussion

Focus group results showed that among attributes participants look at when buying eggs, the free-range and the organic label were the most important ones. Among motivations driving to free-range and/or organic eggs, participants mainly mentioned the perception of higher animal welfare, better taste, better egg quality, higher nutritional content, and high-quality feed. This result is in accordance with studies showing that consumption of free-range and organic eggs, especially in the UK, is mainly related to animal welfare concerns. Moreover, consumers who purchase cage-free eggs also associate the enhanced animal welfare standards with higher quality, safety, and better taste of eggs [37,38].

Although, as stated by Bennett et al. [37] and Pettersson et al. [38], consumers with animal welfare concerns care little about the price of free-range and organic eggs, price in this study was still an important determinant of purchase decision-making, acting as a mediator between production method and affordability. Moreover, based on Fearne and Lavelle [39], UK egg consumers can be segmented into two major clusters according to price and production method. Our study confirmed that although for some participants the production method was more important than price, for others, a low price was the only attribute to consider.

Participants overall showed positive attitudes towards eggs from insect-fed hens [7,9,12,13,15]. In particular, with the provision of information, participants demonstrated that environmental, food waste, and animal welfare benefits positively influenced intentions to consume and purchase the product [5,10,11,12]. In line with the attributes that consumers looked at when purchasing eggs, participants revealed that besides the benefits mentioned, factors such as production method, taste, quality, and nutritional value were important for the evaluation of the product and its potential consumption [4,14,16]. Although many participants believed that by eating insects, hens show higher welfare, they also expected that these eggs should be at least produced and marketed as free-range. Following Bennett et al. [37] and Rondoni, Asioli, and Millan [40], trust in the certification institution was an important factor when evaluating the product.

Price, like certification, was an important determinant for purchasing. As already found in many studies on insect-fed fish, participants were willing to purchase the product [16,17,18,19,20]. However, assessing the price before purchasing appeared to be essential.

Some of the participants revealed disgust and rejection towards insects as feed, demonstrating that this factor may act as a barrier to consumer’s intentions [9,13,14,20]. However, only for very few participants did disgust strongly reduce the intention to purchase the product.

The lack of trust in the benefits of feeding hens with insects appeared to be a potential barrier to acceptance. Consumers had limited knowledge on this product; therefore, they required more detailed information to evaluate it. According to Grunert [41], eco- and animal-friendly food products have attributes of sustainability and animal welfare that can be evaluated by consumers neither before nor after the purchase. Consequently, consumers need to be provided with reliable information to increase their confidence and trust in the product. In line with other studies, the provision of comprehensive information about the benefits of feeding hens with insects may enhance awareness and, in turn, increase consumers’ intentions to consume and purchase the product [13,14,15,20].

Based on our findings, several implications for businesses and stakeholders may be highlighted for the introduction of these eggs to the UK market.

Considering animal welfare concerns, the large market share of free-range eggs, and the importance of labelling accredited by reliable institutions among UK consumers, stakeholders should consider producing and selling eggs from insect-fed hens with enhanced animal welfare standards (e.g., free-range and organic labelling). Insects as feed should be therefore considered more as an additional benefit associated with cage-free eggs (e.g., free-range and organic). In contrast, insect-fed cage hens would be expected to produce too few additional benefits to be accepted and consumed.

The lack of trust in the product and the limited knowledge about insects as feed among consumers suggest that the provision of information about the benefits of feeding hens with insects may increase trust in the product and enhance intentions. However, the negative effect of the word “insects” on the packaging suggests the need to position the product on the market specifying the use of insects as feed but without drawing excessive attention to them on the packaging. In contrast, the positive effect of the word “natural” and the positive attitudes towards benefits highlight the need for companies to position these eggs as a natural, eco- and animal-friendly food product.

The study presents some limitations that should be addressed in future research or a follow-up study. First, due to the restricted sample size, these findings are considered to be exploratory research. Further research is required among a larger sample in order to better define the determinants of consumer acceptance and marketing strategies. Secondly, considering that participants were not provided with information about quality, nutritional content, and taste, further research is recommended in order to understand how the product’s intrinsic attributes may affect consumers’ perceptions. Moreover, price is an important determinant of purchasing. Given the increase in price from the benefits provided, consumers are willing to purchase the product. However, whether consumers are willing to pay a premium price for these eggs needs further investigation.

## 5. Conclusions

This is the first qualitative study exploring consumers’ attitudes and perception towards eggs from insect-fed hens in the UK. It provides preliminary evidence regarding factors affecting acceptance and consumption intentions towards eggs from insect-fed hens.

The study found that UK consumers have positive attitudes towards eggs from insect-fed hens. Consumer acceptance appears to be driven by mainly the environmental, animal welfare, and food waste benefits associated with feeding hens with insects. However, other egg attributes such as price, production method, taste, quality, and nutritional value affect intentions. Price was an important factor when considering a potential purchase. Therefore, whether consumers are willing to pay a premium price for these eggs still needs to be clarified. Although disgust may negatively influence consumer acceptance and intentions towards insects as feed, the lack of awareness about the product and the limited knowledge about its benefits appear to be stronger barriers. With this in mind, our study suggests that educating and informing consumers about the benefits of feeding hens with insects may increase intentions to consume and purchase eggs from insect-fed hens.

The study also suggests the importance of trust in the egg certification on the packaging. Based on the results, we can conclude that replacing soybean meal with insects in hen feed has little effect on consumers’ preferences by itself. In order to encourage consumer consumption, eggs from insect-fed hens should be produced and marketed under enhanced animal welfare standards (e.g., at least free-range labelling).

Despite its exploratory nature, this study contributes to the limited literature on insect-fed foodstuffs. Given the emerging area of research, this study may contribute and pave the way for further qualitative and quantitative studies on the topic.

## Figures and Tables

**Table 1:** Socio-demographics of focus group participants.

Characteristics		Frequency
Gender	Male	9
	Female	10
Age		
	18–24	6
	25–34	8
	Over 34	5
Education level	High School	2
	Bachelor’s degree	9
	Master’s degree	7
	Doctorate	1
Income	Less than £20,000	6
	£20,000 to £39,999	6
	£40,000 to £59,999	4
	£60,000 and higher	3
Diet	Omnivore	16
	Vegetarian	3

In the sample, 18.7% identified themselves as vegetarians. We did not distinguish between more specific dietary patterns such as flexitarian, pescatarian, etc; 18.48% of UK consumers indicate having a diet that is neither “meat eater” nor “vegan” [32], suggesting that vegetarians in the sample can be considered a sufficiently representative sub-subset of the population.

**Table 2:** Focus group structure.

Sections	Questions (sub-sections)
1: Introduction	Introduction of moderator, assistant moderator, and participants. Elucidation of the discussion topic and ground rules. Engagement (ice-braker) question
2: Egg consumption patterns	Exploratory questions (possible use of probing and follow up questions): Egg attributes preferred, and related motivation
3: Attitudes	Provision of information: Card Exploratory questions: Attitudes and perceptions towards eggs from insect-fed hens and factors related
4: Packaging	Provision of information: Packaging Exploratory questions: Reactions and opinions towards the packaging
5: Willingness to buy	Exploratory questions: Willingness to buy Exit questions: Asking for questions and thank participants

**Table 3:** Attitudes and perceptions towards eggs from insect-fed hens.

Main Factors	Main Quotes	Participant
Benefits	*“For me, the fact that you can fed insects with food-by-product is a massive benefit and if it was marketed right, in the same way free-range is marketed, I think lots and lots of people would go for that”* *“I would be encouraged mainly for the environmental benefit and animal welfare aspect. It’s quite an important selling point for me!”* *“I like the fact you can feed eggs with insects because the way in which you feed hens naturally may indirectly help the environment too”*	(P13, F, 25–34 y) (P17, F, over 34 y) (P12, F, 25–34 y)
Lack of trust	*“I’m slightly suspicious they are fed with food waste. It sounds good but again, is it actually like this? I do just not trust so much when it comes to marketing.”* *“My concern is, is a company doing this for improving the system or for their bottom line? It is a cost-cutting or is actually related to the welfare of the hen!?”* *“You are never going to develop a hen feeding system in which there is no impact.”*	(P8, F, 25–34 y) (P11, M, 25–34 y) (P17, F, over 34 y)

**Table 4:** Factors influencing attitudes towards eggs from insect-fed hens.

Main Factors	Main Quotes	Participant
Price	*“Price should be reasonable. If the product costs too much, more than free-range, I wouldn’t give it a go.”*	P1, M, 18–24 y
Information	*“I have to find out more about the product. I have to be sure these benefits exist for sure.”*	P8, F, 25–34 y
Availability	*“For me is the availability on the market the most important point. I can say I would try them, but I don’t think I would go out of my way to buy them.”*	P13, F, 25–34 y
Nutritional content	*“I will be very happy to try these eggs for the fact that insects have good protein level. So, I would not be surprised if research said that eggs fed with insects are better in quality and nutritional content.”*	P17, F, over 34 y
Taste	*“Quality would be at least the same as the others in order for me to buy them. It can be the best product in the world but If it doesn’t taste good, I wouldn’t support them.”*	P4, M, 18–24 y P8, F, 25–34 y
Disgust	*“Taste and nutritional content. These attributes would be important only if you cover the animal welfare and the other issues we discussed.”*	P3, F, 18–24 y

**Table 5:** Reactions and opinions towards the packaging.

Main Factors	Main Quotes	Participant
Disgust	*“I like the message on the back of the packaging, but on the front, the word ‘insect’ is written three times and that kind of puts me off.”*	(P4, M, 18–24 y)
Natural aspect	*“Taste is important. So for me just saying ‘natural taste’ for me is fine, I like it, it’s a good selling point. I’m convinced.”*	(P18, F, over 34 y)
Animal welfare standards	*“There is nothing bad about increasing animal welfare. I still want a free-range egg. I wouldn’t buy eggs from insect-fed caged hens”*	(P5, F, 18–24 y)
Information	*“I would like to see more evidence about the benefits. Why is this product sustainable, etc.?”*	(P1, M, 18–24 y)

**Table 6:** Willingness to buy insect-fed eggs.

Main Factors	Main Quotes	Participant
Price	*“I would probably give this product a go, but I don’t know if I would buy it again. It really depends on the price and how many are in the pack.”* “*It depends on the price. How much do these eggs cost?*”	(P1, M, 18–24 y) (P8, F, 25–34 y)
Disgust	*“I might buy them. The only problem for me is that, there are insects in it!”*	(P3, F, 18–24 y)

## References

[1] Verneau F., La Barbera F., Kolle S., Amato M., Del Giudice T., Grunert K. (2016). The effect of communication and implicit associations on consuming insects: An experiment in Denmark and Italy. Appetite.

[2] Star L., Arsiwalla T., Molist F., Leushuis R., Dalim M., Paul A. (2020). Gradual Provision of Live Black Soldier Fly (Hermetia illucens) Larvae to Older Laying Hens: Effect on Production Performance, Egg Quality, Feather Condition and Behavior. Animals.

[3] Gasco L., Biasato I., Dabbou S., Schiavone A., Sogari G., Mora C., Menozzi D. (2019). Quality and consumer acceptance of products from insect-fed animals. Edible Insects Food Sect.

[4] European Union (2017). Commission Regulation (EU) 2017/1017 of 15 June 2017 amending Regulation (EU) No 68/2013 on the Cat-alogue of feed materials. Off. J. Eur. Union..

[5] Derrien C., Boccuni A., Halloran A., Flore R., Vantomme P., Roos N. (2018). Current status of the insects producing industry in Europe. Edible Insects in Sustainable Food Systems.

[6] Insect Biomass Task, Group F (2019). The Insect Biomass Industry for Animal Feed—The Case for UK-based and Global Business. https://www.fera.co.uk/media/wysiwyg/Final_Insect_Biomass_TF_Paper_Mar19.pdf.

[7] Verbeke W., Spranghers T., De Clercq P., De Smet S., Sas B., Eeckhout M. (2015). Insects in animal feed: Acceptance and its determinants among farmers, agriculture sector stakeholders and citizens. Anim. Feed. Sci. Technol..

[8] Laureati M., Proserpio C., Jucker C., Savoldelli S. (2016). New sustainable protein sources: Consumers’ willingness to adopt insects as feed and food. Ital. J. Food Sci..

[9] Kostecka J., Konieczna K., Cunha L. (2017). Evaluation of insect-based food acceptance by representatives of Polish consumers in the context of natural resources processing retardation. J. Ecol. Eng..

[10] Onwezen M., Puttelaar J.V.D., Verain M., Veldkamp T. (2019). Consumer acceptance of insects as food and feed: The relevance of affective factors. Food Qual. Prefer..

[11] Sogari G., Amato M., Biasato I., Chiesa S., Gasco L. (2019). The Potential Role of Insects as Feed: A Multi-Perspective Review. Animals.

[12] De Faria Domingues C.H., Rossi Borges J.A., Ruviaro C.F., Freire Guidolin D.G., Mauad Carrijo J.R. (2020). Understanding the factors influencing consumer willingness to accept the use of insects to feed poultry, cattle, pigs and fish in Brazil. PLoS ONE.

[13] Roma R., Palmisano G.O., De Boni A. (2020). Insects as Novel Food: A Consumer Attitude Analysis through the Dominance-Based Rough Set Approach. Foods.

[14] Szendrő K., Nagy M.Z., Tóth K. (2020). Consumer Acceptance of Meat from Animals Reared on Insect Meal as Feed. Animals.

[15] Naranjo-Guevara N., Fanter M., Conconi A.M., Floto-Stammen S. (2021). Consumer acceptance among Dutch and German students of insects in feed and food. Food Sci. Nutr..

[16] Mancuso T., Baldi L., Gasco L. (2016). An empirical study on consumer acceptance of farmed fish fed on insect meals: The Italian case. Aquac. Int..

[17] Popoff M., MacLeod M., Leschen W. (2017). Attitudes towards the use of insect-derived materials in Scottish salmon feeds. J. Insects Food Feed..

[18] Ankamah-Yeboah I., Jacobsen J.B., Olsen S.B. (2018). Innovating out of the fishmeal trap: The role of insect-based fish feed in consumers’ preferences for fish attributes. Br. Food J..

[19] Ferrer Llagostera P., Kallas Z., Reig L., Amores de Gea D. (2019). The use of insect meal as a sustainable feeding alternative in aqua-culture: Current situation, Spanish consumers’ perceptions and willingness to pay. J. Clean Prod..

[20] Bazoche P., Poret S. (2020). Acceptability of insects in animal feed: A survey of French consumers. J. Consum. Behav..

[21] Krueger R.A., Casey M.A. (2000). Focus Groups: A Practical Guide for Applied Research.

[22] Rabiee F. (2004). Focus-group interview and data analysis. Proc. Nutr. Soc..

[23] Sogari G. (2015). Entomophagy and Italian consumers: An exploratory analysis. Prog. Nutr..

[24] Tan H.S.G., Fischer A.R., Tinchan P., Stieger M., Steenbekkers L., Van Trijp H.C. (2015). Insects as food: Exploring cultural exposure and individual experience as determinants of acceptance. Food Qual. Prefer..

[25] Balzan S., Fasolato L., Maniero S., Novelli E. (2016). Edible insects and young adults in a north-east Italian city an exploratory study. Br. Food J..

[26] Pambo K., Mbeche R., Okello J., Kinyuru J., Mose G. (2016). Consumers’ salient beliefs regarding foods from edible insects in Kenya: A qualitative study using concepts from the theory of planned behaviour. Afr. J. Food, Agric. Nutr. Dev..

[27] Marberg A., Van Kranenburg H., Korzilius H. (2017). The big bug: The legitimation of the edible insect sector in the Netherlands. Food Policy.

[28] Sogari G., Menozzi D., Mora C. (2017). Exploring young foodies׳ knowledge and attitude regarding entomophagy: A qualitative study in Italy. Int. J. Gastron. Food Sci..

[29] Clarkson C., Mirosa M., Birch J. (2018). Consumer acceptance of insects and ideal product attributes. Br. Food J..

[30] Myers G., Pettigrew S. (2018). A qualitative exploration of the factors underlying seniors’ receptiveness to entomophagy. Food Res. Int..

[31] Sogari G., Bogueva D., Marinova D. (2019). Australian Consumers’ Response to Insects as Food. Agriculture.

[32] Statista. https://www-statista-com.eu1.proxy.openathens.net/statistics/1066772/main-dietary-habits-in-the-united-kingdom/.

[33] (2018). NVivo Qualitative Data Analysis Software.

[34] Nowell L.S., Norris J.M., White D.E., Moules N.J. (2017). Thematic Analysis: Striving to Meet the Trustworthiness Criteria. Int. J. Qual. Methods.

[35] Gale N.K., Heath G., Cameron E., Rashid S., Redwood S. (2013). Using the framework method for the analysis of qualitative data in multi-disciplinary health research. BMC Med Res. Methodol..

[36] Fereday J., Muir-Cochrane E. (2006). Demonstrating rigor using thematic analysis: A hybrid approach of inductive and deductive coding and theme development. Int. J. Qual. Methods.

[37] Bennett R., Jones P., Nicol C., Tranter R., Weeks C.A. (2016). Consumer attitudes to injurious pecking in free-range egg production. Anim. Welf..

[38] Pettersson I.C., Weeks C.A., Wilson L.R.M., Nicol C.J. (2016). Consumer perceptions of free-range laying hen welfare. Br. Food J..

[39] Fearne A., Lavelle D. (1996). Segmenting the UK egg market: Results of a survey of consumer attitudes and perceptions. Br. Food J..

[40] Rondoni A., Asioli D., Millan E. (2020). Consumer behaviour, perceptions, and preferences towards eggs: A review of the literature and discussion of industry implications. Trends Food Sci. Technol..

[41] Grunert K.G. (2011). Sustainability in the Food Sector A Consumer Behaviour Perspective. Int. J. Food Syst. Dyn..

